# Protective Effect of Indole-3-Pyruvate against Ultraviolet B-Induced Damage to Cultured HaCaT Keratinocytes and the Skin of Hairless Mice

**DOI:** 10.1371/journal.pone.0096804

**Published:** 2014-05-08

**Authors:** Reiji Aoki, Ayako Aoki-Yoshida, Chise Suzuki, Yoshiharu Takayama

**Affiliations:** 1 Functional Biomolecules Research Group, National Agriculture and Food Research Organization, Tsukuba, Ibaraki, Japan; 2 Department of Applied Biological Chemistry, Graduate School of Agricultural and Life Sciences, The University of Tokyo, Bunkyo-ku, Tokyo, Japan; University of Tennessee, United States of America

## Abstract

Previous investigations demonstrated that pyruvate protects human keratinocytes against cell damage stemming from exposure to ultraviolet B (UVB) radiation. This study endeavoured to elucidate the protective capacity of aromatic pyruvates (e.g., phenylpyruvate (PPyr), 4-hydroxyphenylpyruvate (HPPyr), and indole-3-pyruvate (IPyr)) against UVB-induced injury to skin cells, both *in vitro* and *in vivo*. Cultured human HaCaT keratinocytes were irradiated with UVB light (60 mJ/cm^2^) and maintained with or without test compounds (1–25 mM). In addition, the dorsal skin of hairless mice (HR-1) was treated with test compounds (100 µmol) and exposed to UVB light (1 J/cm^2^) for two times. The ability of the test compounds to ameliorate UVB-induced cytotoxicity and inflammation was then assessed. Aromatic pyruvates reduced cytotoxicity in UVB-irradiated HaCaT keratinocytes, and also diminished the expression of interleukin 1β (IL-1β) and interleukin 6 (IL-6). IPyr was more efficacious than either PPyr or HPPyr. Furthermore, only IPyr inhibited cyclooxygenase-2 (Cox-2) expression at both the mRNA and the protein level in UVB-treated keratinocytes. Topical application of IPyr to the dorsal skin of hairless mice reduced the severity of UVB-induced skin lesions, the augmentation of dermal thickness, and transepithelial water loss. Overproduction of IL-1β and IL-6 in response to UVB radiation was also suppressed *in vivo* by the topical administration of IPyr. These data strongly suggest that IPyr might find utility as a UVB-blocking reagent in therapeutic strategies to lessen UVB-induced inflammatory skin damage.

## Introduction

Ultraviolet B (UVB) radiation causes serious injury to skin cells and tissues (and especially to the epidermis) by inducing the production of inflammatory mediators and DNA lesions and, ultimately, by provoking cellular apoptosis.[1], [2] Reactive oxygen species (ROS) play important roles in the cascade of events preceding UV/UVB-induced inflammation and apoptosis in the skin,[1], [2] while natural antioxidants, including carotenoids, glutathione, vitamin C (ascorbic acid), and vitamin E (α-tocopherol), prevent or lessen UVB-induced skin damage.[1], [3], [4].

Recent findings indicate that skin is a peripheral neuroendocrine organ. Mammalian skin expresses components of hypothalamic pituitary adrenal (HPA) axis, including corticoprotein releasing hormone (CRF), proopiomelanocortin (POMC), and cortisol/corticosterone [5]. In response to UVB-irradiation, epidermal and dermal cells produce stress neurotransmitters, neuropeptides and hormones [5], [6]. Increased expression of HPA axis gene is observed in UVB-irradiated skin or co-culture model using keratinocyte and melanocyte [6], [7].

We previously demonstrated that pyruvate, the end product of glycolysis, contributes to the prevention of UVB-provoked cytotoxicity and the generation of certain pro-inflammatory mediators in immortalised human HaCaT keratinocytes.[8] Pyruvate also acts as an endogenous antioxidant and has the ability to suppress inflammation and organ dysfunction caused by injury.[9] However, the antioxidant activity of pyruvate is not entirely responsible for its anti-inflammatory properties. Indeed, we recently showed that pyruvate failed to inhibit the production of ROS in UVB-irradiated HaCaT keratinocytes,[8] indicating that the antioxidant capabilities of the compound are at least partially distinct from its anti-inflammatory actions.

In addition to pyruvate itself, various pyruvate derivatives can reduce inflammatory responses triggered by tissue injury. For example, ethyl pyruvate (EP) is a lipophilic derivative of pyruvate that exerts cytoprotective actions against numerous pathological conditions (e.g., mesenteric ischemia, acute endotoxemia, bacterial peritonitis, and tumour angiogenesis) by virtue of its anti-inflammatory characterisitics.[9], [10] On the other hand, assorted indole derivatives, such as melatonin and indole-3-lactic acid (ILA), function to reduce cytotoxicity and/or the production of pro-inflammatory regulators in UVB-irradiated keratinocytes.[11], [12], [13], [14], [15] Melatonin acts multiple ways for preventing skin photodamage. Induction of antioxidant enzymes is dependent on signal transduction pathways related to melatonin receptors. High-affinity G-protein coupled melatonin receptors (MT1 and MT2) were found in cultured human skin cells. In addition, the nuclear receptor retinoic acid orphan receptor type α (RORα) is known as a mediator of nuclear melatonin signaling.[16], [17] Direct antioxidant and radical scavenging activities are receptor-independent action of melatonin. [16], [17]. This suggests that an indole derivative of pyruvate might be particularly efficacious in the pharmacological arsenal against UVB-induced skin injury.

Indole-3-pyruvate (IPyr) is the keto-analogue of tryptophan, and is recognised as an intermediate in the metabolism of tryptophan to ILA or indole-3-acetate (IAA). Administration of IPyr reduced anxiety in human patients in a clinical trial, although its mechanism of action was not established.[18] IPyr also attenuated oxidative cellular injury in the brain, but the compound was much less effective at preventing oxidative stress compared with other indole derivatives, such as indole-3-propionate. [19], [20].

The purpose of this study was to examine whether IPyr could prevent or down-regulate the inflammatory response and damage to the skin consequent to UVB irradiation in cultured HaCaT keratinocytes and HR-1 hairless mice. The skin-protective capacity of IPyr relative to pyruvate and other types of aromatic pyruvates (i.e., 3-phenylpyruvate (PPyr) and 4-hydroxyphenyl pyruvate (HPPyr)) was also investigated.

## Materials and Methods

### Preparation of Test Compounds

For the *in vitro* study, pyruvic acid (Wako Pure Chemical, Osaka, Japan), PPyr (Wako), HPPyr (Sigma-Aldrich, St Louis, MO, USA), and IPyr (Sigma-Aldrich) were dissolved in Dulbecco’s Modified Eagle’s Medium (DMEM; Sigma-Aldrich) and neutralised with an equimolar amount of sodium hydroxide (NaOH). For the *in vivo* study, PPyr, HPPyr, and IPyr were dissolved in 0.05 M phosphate buffer (pH 7.0) containing 30% propylene glycol and 20% ethanol, and neutralised by the addition of 2N NaOH.

### Animals

Male HR-1 hairless mice (8 weeks old at experimental onset; Charles River Japan, Kanagawa, Japan) were employed in this study. Mice were allowed to acclimate for 1 week, with free access to standard rodent chow (MM-3; Funabashi Farm, Chiba, Japan) and water. All animal care and use protocols were conducted in accordance with the animal experimentation guidelines of the National Agriculture and Food Research Organization (NARO; Ibaraki, Japan). The protocol was approved by the Animal Care Committee, National Institute of Livestock and Grassland Science (Ibaraki, Japan; permit number: 13033154).

### Cell Culture

HaCaT keratinocytes (Cell Lines Service, Eppelheim, Germany) were maintained in high-glucose DMEM supplemented with 10% fetal bovine serum, 100 U/ml penicillin (Life Technologies, Carlsbad, CA, USA), and 100 U/ml streptomycin (Life Technologies) in an atmosphere of 5% CO_2_/95% air at 37°C.

### UVB Irradiation of HaCaT Keratinocytes

HaCaT keratinocytes were exposed to UVB radiation, as described previously.[8] Briefly, the cells were grown to confluence prior to UVB irradiation, and the culture medium was replaced with Hank’s Balanced Salt Solution (HBSS; Life Technologies). A UVB lamp (Model G8T5E, Sankyo Denki, Kanagawa, Japan) with an energy spectrum of 280–320 nm and a maximum emission at 306 nm was used as the UVB light source. UVB exposure was monitored by using a UV-340A light meter (Lutron, Taipei, Taiwan). Following UVB treatment, the spent HBSS was replaced with fresh culture growth medium with or without test compounds for the indicated periods of time.

### Cell Viability Assays

The number of viable HaCaT cells in each culture was estimated by using the crystal violet survival/proliferation assay, as described previously.[21] HaCaT cells were fixed with 3.7% formaldehyde in phosphate buffered saline (PBS) for 10 min and washed twice with PBS. The cells were then stained with 0.1% crystal violet (Merck, Darmstadt, Germany) in PBS. After two washes with PBS, the dye was eluted with 10% acetic acid (2 ml), and the absorbance at 595 nm was measured.

### Cytokine Production Assay

The production of interleukin 1β (IL-1β) and interleukin 6 (IL-6) in the conditioned culture medium was measured by the conventional enzyme-linked immunosorbent assay (ELISA) procedure by using the Opt EIA ELISA Kit for human IL-1β and IL-6 (BD Bioscience, San Diego, CA, USA) according to the manufacturer’s protocol.

### Western Blotting Assay

HaCaT cells were extracted with RIPA buffer (Nacalai Tesque, Kyoto, Japan) to yield cell lysates. To determine the protein expression of cyclooxygenase-2 (Cox-2) and the phosphorylation status of p38 mitogen-associated protein kinase (p38 MAPK; Thr180/Tyr182) in the cells, cell lysates were resolved by 10% sodium dodecyl sulphate polyacrylamide gel electrophoresis (SDS-PAGE) according to the method of Laemmli, followed by Western blotting analysis with anti-Cox-2 and anti-phosphpo p38 MAPK (p-p38 MAPK) antibodies, as described previously.[8] Glyceraldehyde 3-phosphate dehydrogenase (GAPDH) and total p38 MAPK served as the loading controls.

### UVB Irradiation of HR-1 Hairless Mice

HR-1 mice were randomly divided into five groups (sham-irradiated control, UVB+solvent, UVB+PPyr, UVB+HPPyr, and UVB+IPyr; n = 8 animals per group). The dorsal skin of each mouse was treated with test compounds (100 µmol) or with the solvent control. The dorsal skin was exposed to UVB radiation by using an EL Series UVB Lamp (Model UVLM-28; UVP, Upland, CA, USA) with maximum emission at 302 nm (Fig. S1A). The Lamp was equipped with a filter to cut off wavelengths below 290 nm. Intensity of UVB was 1,000 µW/cm^2^ (monitored by a UV-340A light meter), and the dose was 1 J/cm^2^. This dose was achieved after 1,000 s irradiation time. During the UVB exposure, the mice were housed in specially designed cages, where they were held in individual dividers separated by stainless steel gauze (Fig. S1B). The mice were not anesthetized at any time during the radiation exposure. UVB-irradiation was performed on days 1 and 3 of the experiment. Transepithelial water loss was measured by using a Tewameter TM-300MP device (Courage and Khazaka, Köln, Germany) according to the manufacturer’s protocol. On day 5, the mice were killed by cervical dislocation. For analysis of Bax (B-cell lymphoma 2 (Bcl-2)-associated X protein), Cox-2, IL-1β, and IL-6 mRNA expression, dorsal skin biopsy samples were stored in RNAlater solution (Life Technologies) for RNA stabilisation and preservation. For microscopic evaluation, skin biopsy samples were fixed with 3.7% formaldehyde in PBS, embedded in paraffin, sectioned at a thickness of 5 µm, and stained with hematoxylin and eosin. Images were taken by using an Axioplan 2 upright microscope equipped with an Axiocam digital camera (Carl Zeiss, Göttingen, Germany).

### Real-time Polymerase Chain Reaction (PCR)

Total RNA derived from HaCaT keratinocytes and dorsal mouse skin was isolated by using an RNeasy Mini Kit (Qiagen, Hilden, Germany). Total RNA was reverse-transcribed with the ReverTra Ace qPCR RT Master Mix (Toyobo, Osaka, Japan), and real-time PCR was performed by using a T100 Thermal Cycler (Bio-Rad, Hercules, CA, USA) and the Thunderbird SYBR qPCR Mix (Toyobo) according to the manufacturer’s protocol. All primer sequences employed in this study are shown in Table S1.

### Statistical Analysis

For the *in vitro* study, the mean value of each group was compared by using Tukey’s multiple comparison test (*P*<0.05). For the *in vivo* study, the statistical significance between two groups was evaluated by using Student’s t-test (*P*<0.05).

## Results

### Aromatic Pyruvates Improve the Survival Rate of UVB-irradiated HaCaT Keratinocytes

**Figure 1:**
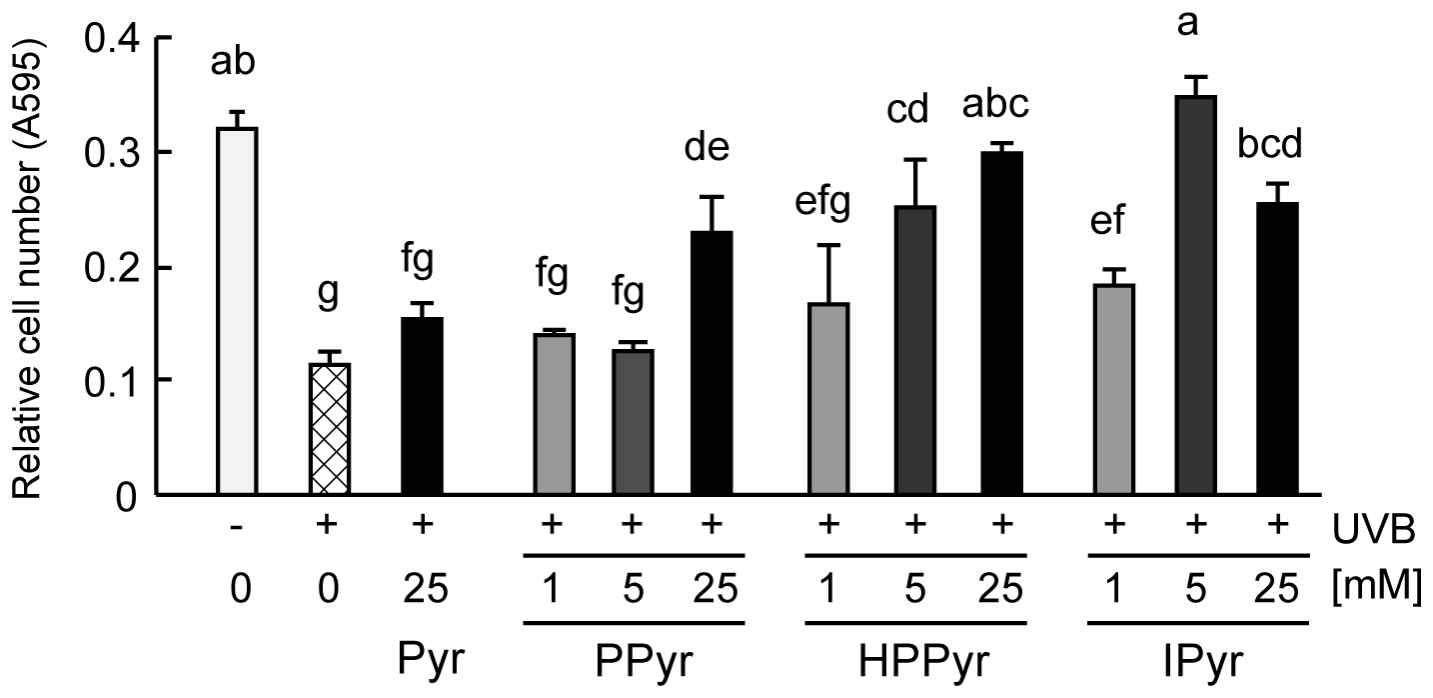
Aromatic pyruvates protect against ultraviolet B (UVB)-induced cytotoxicity *in vitro*. Following UVB irradiation (60 mJ/cm^2^), HaCaT cells were treated for 24 h with solvent (culture medium) or with the indicated concentrations of pyruvate (Pyr), phenylpyruvate (PPyr), 4-hydroxyphenyl pyruvate (HPPyr), or indole-3-pyruvate (IPyr). Control, non-irradiated cells were treated with solvent alone. The number of attached, viable cells was then estimated by crystal violet staining. Bars and error bars represent the means and standard deviations for three independent measurements of the absorbance at 595 nm (A595). Bars with the same letter represent data that do not differ significantly from each other (Tukey’s test, *P*<0.05).

We first performed a crystal violet assay to evaluate the cytoprotective effects of aromatic pyruvates against UVB-induced cell damage to keratinocytes, the predominant cell type found in the epidermis. As shown in Figure 1, UVB irradiation significantly decreased the number of attached, viable HaCaT cells compared with the non-irradiated group, but supplementation of the culture medium with pyruvate (Pyr, 25 mM) or the aromatic pyruvates (25 mM) for 24 h significantly increased cell numbers in the irradiated cultures. All of the aromatic pyruvates were significantly more effective at 25 mM than pyruvate itself. IPyr significantly reduced the cytotoxic effect of UVB at the lowest concentration employed (1 mM), whereas the other aromatic pyruvates did not.

### Aromatic Pyruvates Inhibit UVB-induced Production of Inflammatory Cytokines

**Figure 2:**
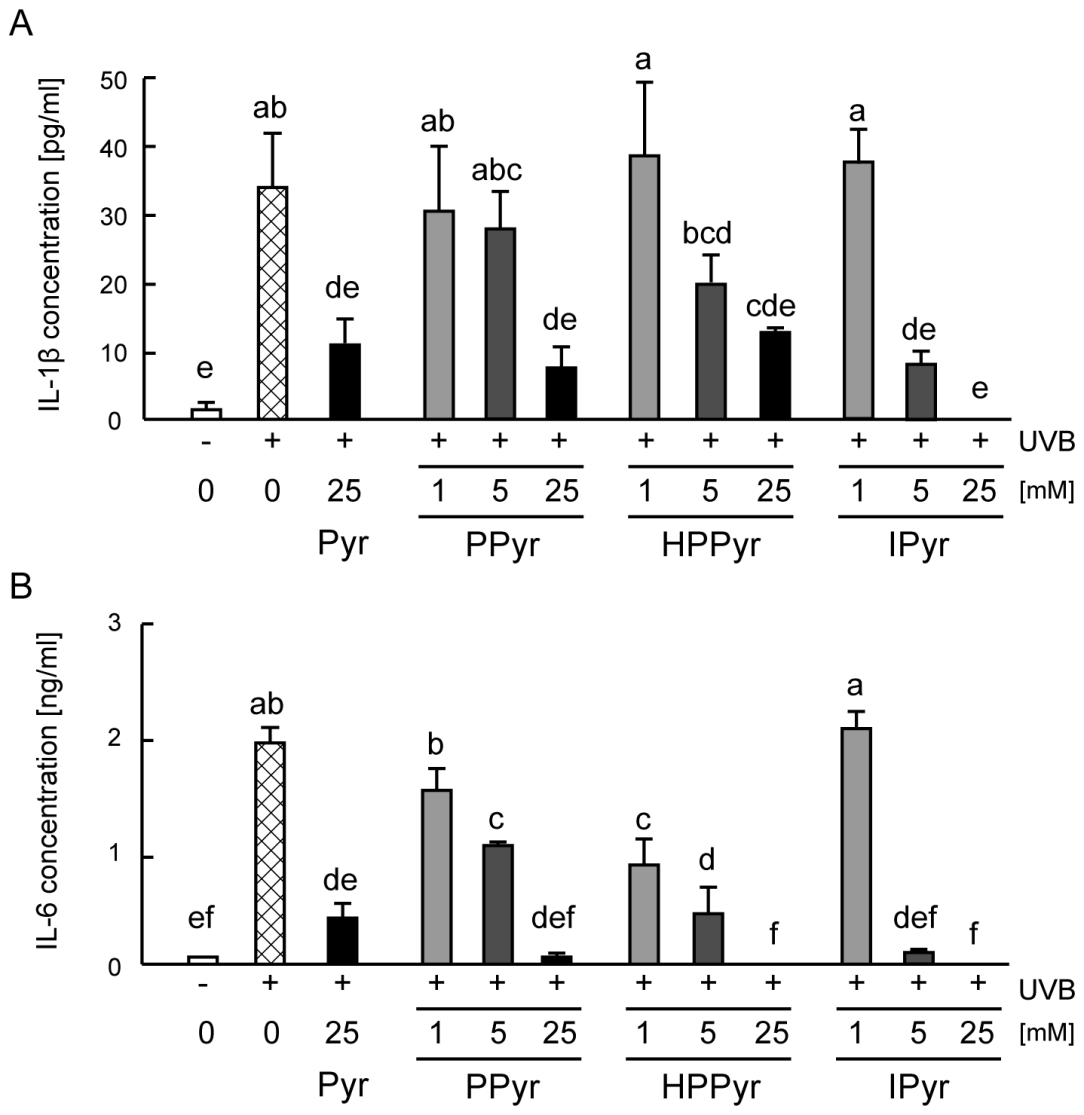
Aromatic pyruvates protect against UVB-induced secretion of interleukin 1β (IL-1β) and interleukin 6 (IL-6). Following UVB irradiation (60 mJ/cm^2^), HaCaT cells were treated with solvent (culture medium) or with the indicated concentrations of Pyr, PPyr, HPPyr, or IPyr for 24 h. Control, non-irradiated cells were treated with solvent alone. An enzyme-linked immunosorbent assay (ELISA) analysis was used to estimate the concentrations of secreted IL-1β (A) and IL-6 (B). Bars and error bars represent the means and standard deviations for three independent measurements. Bars with the same letter represent data that do not differ significantly from each other (Tukey’s test, *P*<0.05).

**Figure 3:**
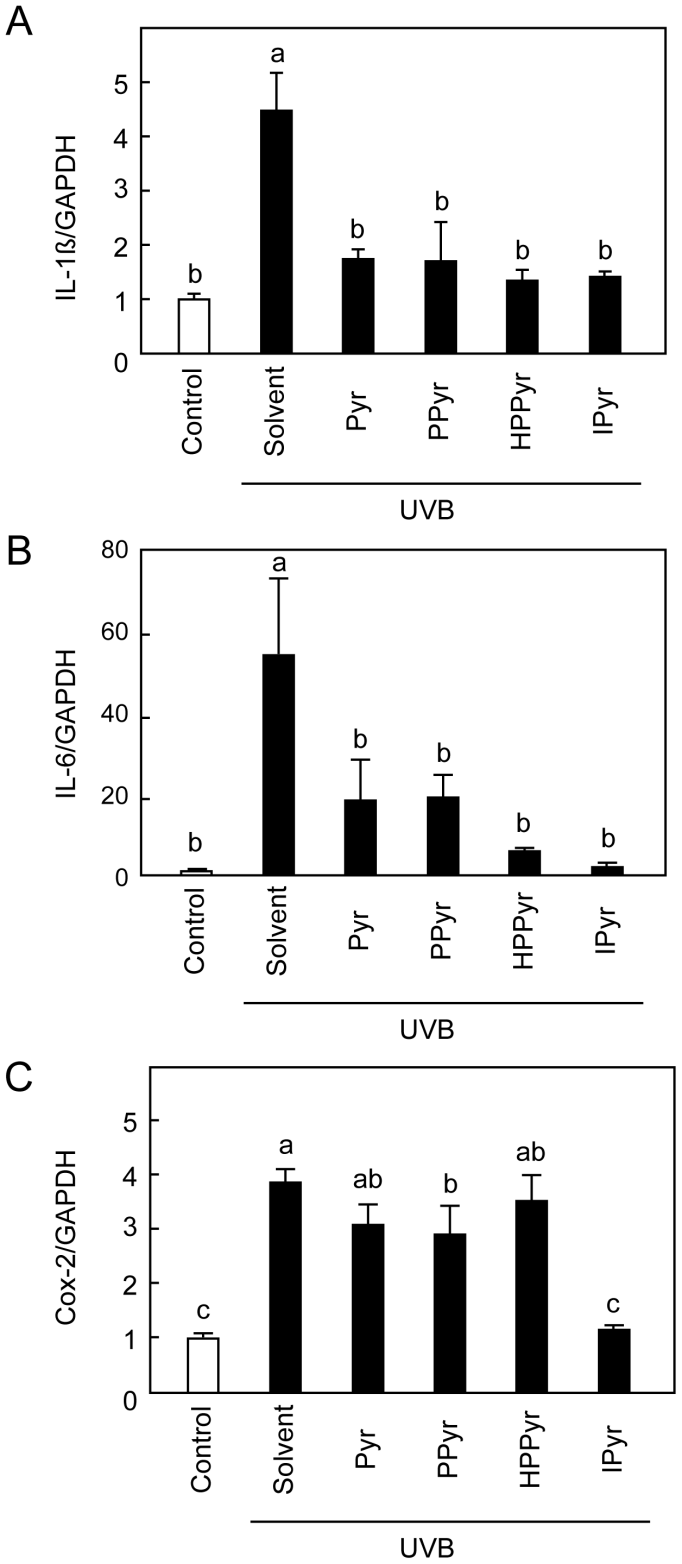
Aromatic pyruvates inhibit UVB-stimulated mRNA expression of IL-1β, IL-6, and cyclooxygenase 2 (Cox-2) in keratinocytes. Following UVB irradiation (60 mJ/cm^2^), HaCaT cells were treated with solvent (culture medium) or with 25 mM Pyr, PPyr, HPPyr, or IPyr for 6 h. Control, non-irradiated cells were treated with solvent alone. The mRNA expression levels of IL-1β (A), IL-6 (B), and Cox-2 (C) were measured relative to that of glyceraldehyde 3-phosphate (GAPDH) by real-time PCR. Bars and error bars represent the means and standard deviations for three independent measurements. Bars with the same letter represent data that do not differ significantly from each other (Tukey’s test, *P*<0.05).

To analyse whether the aromatic pyruvates could inhibit UVB-induced inflammatory responses in cultured keratinocytes, secretion of IL-1β and IL-6 into the conditioned culture medium was next measured following the exposure of HaCaT cells to UVB light. UVB irradiation significantly enhanced the levels of secreted IL-1β and IL-6 at 24 h after exposure (Fig. 2A, B). However, this action was dose-dependently suppressed by PPyr, HPPyr, and IPyr. In the presence of 25 mM IPyr, secreted IL-1β (Fig. 2A) and IL-6 (Fig. 2B) levels were comparable to those found in the non-irradiated cells. Pyruvate (25 mM), PPyr (5–25 mM), HPPyr (1–25 mM), and IPyr (5–25 mM) significantly attenuated the UVB-stimulated secretion of IL-6 to a similar extent (Fig. 2B), but IPyr tended to be more efficacious at preventing IL-1β secretion (Fig. 2B). The inhibitory effects of the aromatic pyruvates were confirmed at the mRNA level by real-time PCR. Whereas UVB irradiation increased the mRNA expression levels of IL-1β and IL-6 in solvent (culture medium)-treated HaCaT cells relative to the non-irradiated control, pyruvate and all examined aromatic pyruvates (25 mM) prevented this increase (Fig. 3A, B).

### IPyr Inhibits UVB-induced Production of Cox-2

**Figure 4:**
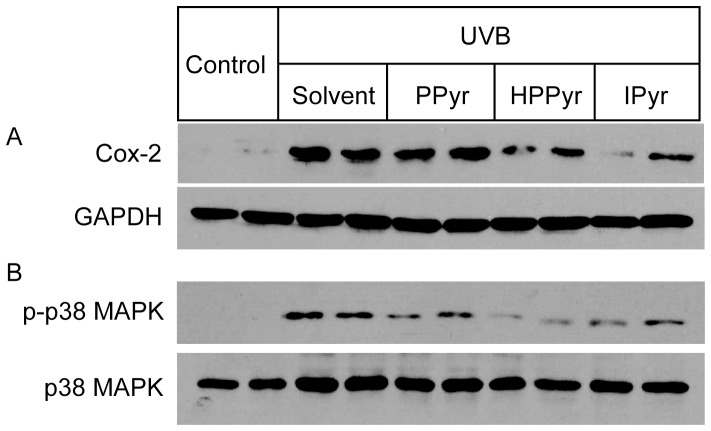
Aromatic pyruvates inhibit UVB-provoked induction of Cox-2 expression and activation of p38 MAPK in keratinocytes. Following UVB irradiation (60 mJ/cm^2^), HaCaT cells were treated with solvent (culture medium) or with 5 mM PPyr, HPPyr, or IPyr. Control, non-irradiated cells were treated with solvent alone. Western blotting analysis of duplicate samples shows Cox-2 protein expression (A) and p38 MAPK phosphorylation (B) in the cells under the various experimental conditions. GAPDH and total p38 MAPK served as the loading controls.

Cox-2 plays key roles in the acute inflammatory response in UVB-irradiated keratinocytes.[22] To further evaluate the anti-inflammatory efficacy of the aromatic pyruvates, we next compared the impact of pyruvate, PPyr, HPPyr, and IPyr (25 mM) on UVB-induced Cox-2 mRNA expression levels by real-time PCR analysis at 6 h after exposure (Fig. 3C). Among the tested compounds, only IPyr significantly inhibited Cox-2 mRNA expression. IPyr also down-regulated Cox-2 expression at the protein level, as shown by Western blotting analysis (Fig. 4A), while PPyr and HPPyr were less effective.

UVB irradiation of epidermal keratinocytes results in the activation of the p38 MAPK signalling pathway, where p38 MAPK is a critical regulator of UVB-induced inflammatory responses and subsequent apoptotic cell death.[1], [23], [24] To elucidate the molecular mechanism underlying the inhibitory effect of the aromatic pyruvates against UVB-induced production of inflammatory cytokines and Cox-2, we next investigated the ability of PPyr, HPPyr, and IPyr to interfere with the UVB-induced phosphorylation of p38 MAPK at 1 h time point. As shown in Figure 4B, the UVB-induced augmentation of p38 MAPK phosphorylation levels was attenuated by all examined aromatic pyruvates, especially HPPyr and IPyr.

### Effects of Aromatic Pyruvates on UVB-stimulated ROS Production

We next explored whether the protective effects of the aromatic pyruvates against UVB irradiation resulted from their antioxidant activity. To do this, we evaluated the impact of PPyr, HPPyr, and IPyr on ROS levels in UVB-irradiated HaCaT cells (Fig. S2). ROS content was markedly increased at 30 min after exposure to UVB light. However, treatment of the UVB-irradiated cells with PPyr, HPPyr, or IPyr failed to inhibit ROS production.

### IPyr Protects Against UVB-induced Skin Damage in HR-1 Hairless Mice

**Figure 5:**
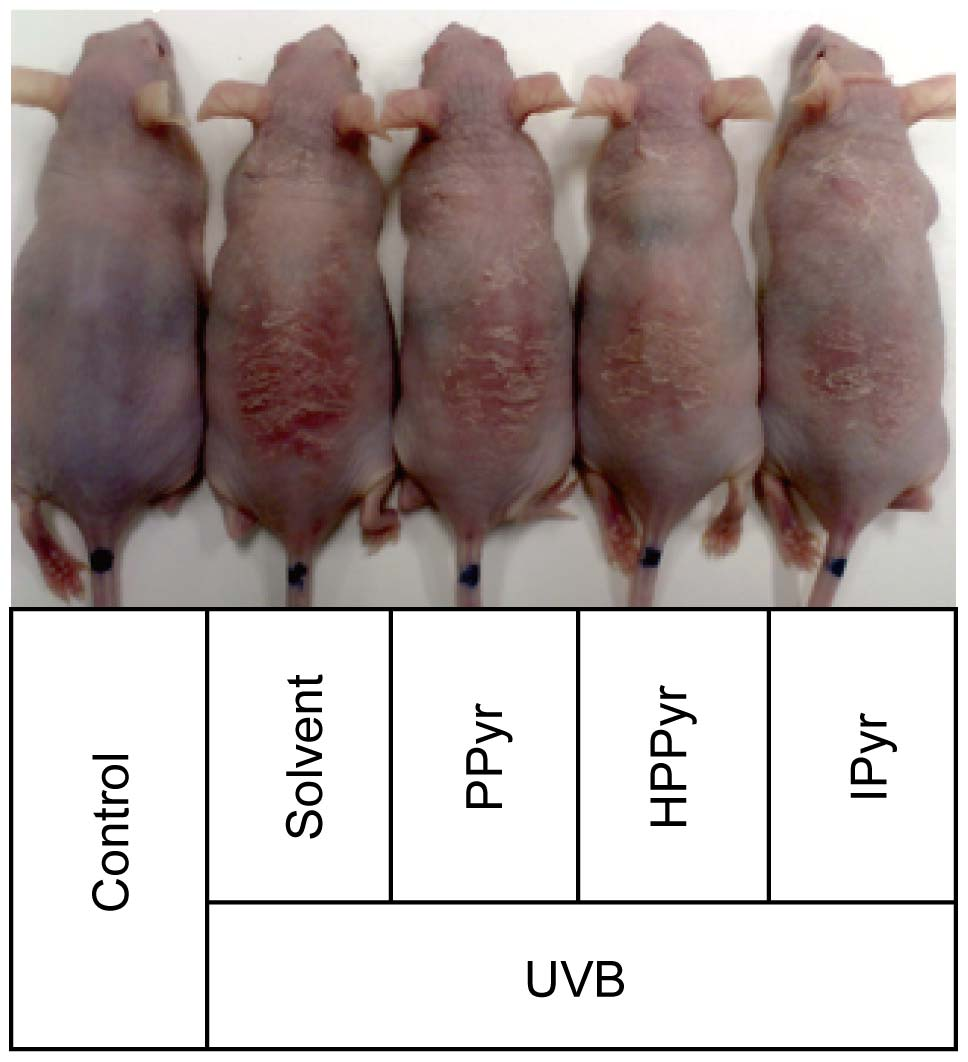
Aromatic pyruvates protect against UVB-induced erythema in HR-1 hairless mice. The dorsal skin surfaces of the hairless mice were treated with solvent (0.05 M) phosphate buffer (pH 7.0) containing 30% propylene glycol and 20% ethanol, PPyr, HPPyr, or IPyr (100 µmol), and subjected to UVB irradiation (1 J/cm^2^). A representative image is shown that demonstrates the appearance of the dorsal skin area in sham-irradiated (control) and UVB-irradiated mice with and without aromatic pyruvates at the end of the experiment (day 5).

The protective role of the aromatic pyruvates against UVB-induced damage was next investigated in HR-1 hairless mice (Fig. 5). Following the exposure of the dorsal skin of these mice to UVB light, skin lesions (evidenced on day 5 as erythema) were observed in all irradiated animal groups by gross exmaination, but the lesions were less severe in PPyr-, HPPyr-, and IPyr-treated, UVB-irradiated mice compared with solvent-treated, UVB-irradiated mice (Fig. 5). No skin lesions were observed at the same time point for the control group (sham-irradiated, solvent-treated mice) (Fig. 5). The beneficial impact of IPyr was more pronounced at the gross morphological level than that of either PPyr or HPPyr.

**Figure 6:**
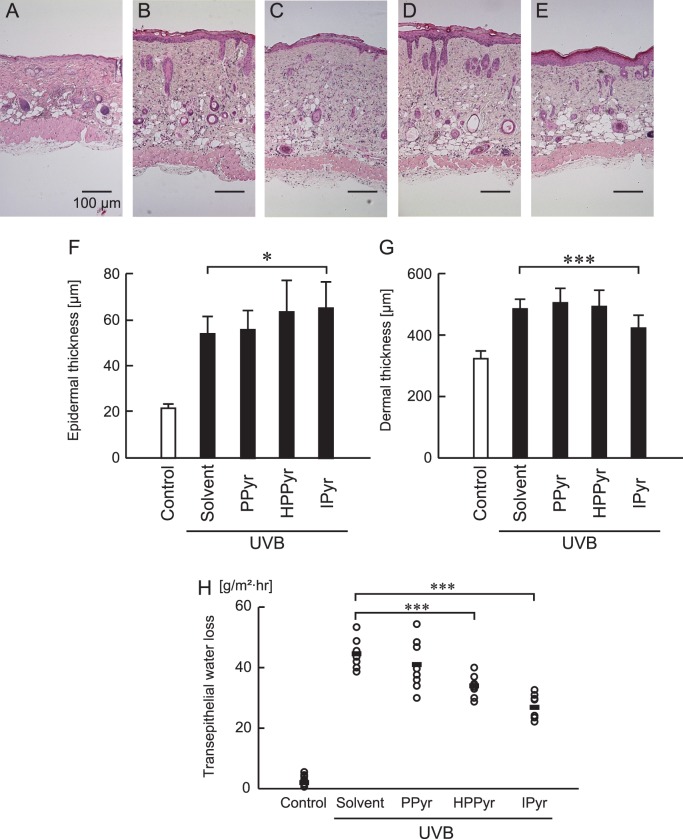
IPyr diminishes UVB-induced dermal thickness in HR-1 hairless mice. (A–E) Hematoxylin and eosin staining of the dorsal skin from sham-irradiated (control) (A) or UVB-irradiated (B–E) hairless mice treated with solvent (B), PPyr (C), HPPyr (D), or IPyr (E). Scale bars, 100 µm. (F, G) Epidermal (F) and dermal (G) thicknesses were measured at the end of the experiment (day 5). Bars and error bars indicate the means and standard deviations for three independent measurements. The statistical significance between the IPyr-treated and the solvent-treated group is indicated as *P<0.05 (F) or ***P<0.001 (G). (H) Transepithelial water loss was measured at the end of experiment (day 5). The statistical significance between the IPyr or HPPyr-treated group and the solvent-treated group is indicated as ***P<0.001.

Because UVB irradiation affects the structure of the skin, we also assessed the ability of the aromatic pyruvates to alter the morphology of the irradiated dermis and the epidermis. The thickness of both compartments was increased in UVB-irradiated mice (Fig. 6B–E) compared with the sham-irradiated control (Fig. 6A), indicative of structural injury. Unexpectedly, IPyr treatment further enhanced the epidermal thickness in UVB-irradiated mice relative to solvent-treated, UVB-irradiated mice (Fig. 6A, E, F). However, the compound significantly reversed the UVB-induced increase in dermal thickness (Fig. 6G). Treatment with PPyr or HPPyr did not significantly affect either epidermal or dermal thickness in the irradiated mice. High-level UVB exposure leads to diffuse cellular damage and eventual tissue necrosis. We found that UVB-induced necrosis observed in upper layer of dermis was attenuated in IPyr-treated mice (Fig. 6E).

In addition, we examined skin barrier disruption following UVB treatment by assessing transepithelial water loss. Transepithelial water loss was significantly higher in UVB-irradiated versus sham-irradiated control mice, and significantly lower in HPPyr- and IPyr-treated, UVB-irradiated mice versus the solvent-treated, UVB-irradiated group (Fig. 6H). These results suggest that both HPPyr and IPyr can prevent the UVB-induced destruction of the skin barrier.

### IPyr Attenuates UVB-provoked Pro-inflammatory and Pro-apoptotic Responses *in vivo*

**Figure 7:**
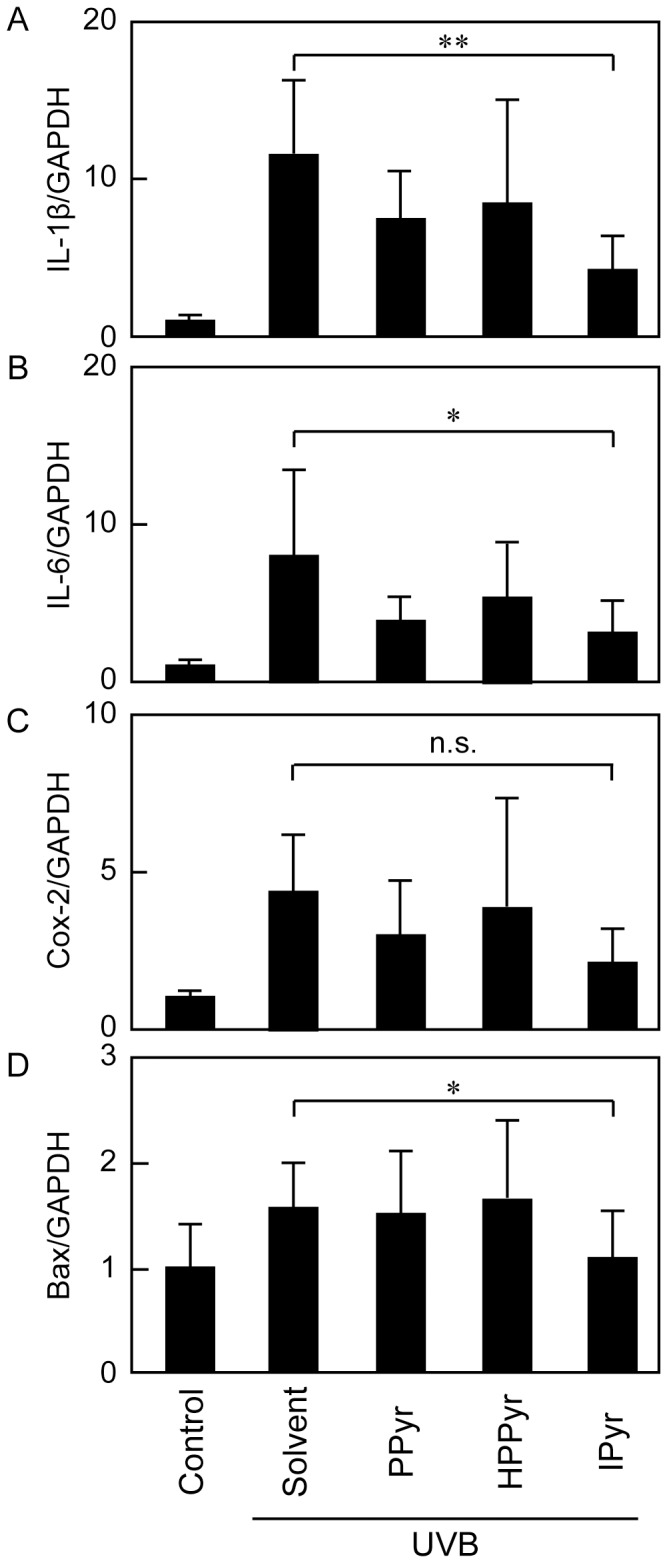
IPyr down-regulates UVB-induced mRNA expression of IL-1β, IL-6, Cox-2, and Bax in HR-1 hairless mice. The mRNA levels of IL-1β (A), IL-6 (B), Cox-2 (C), and B-cell lymphoma 2 (Bcl-2)-associated X protein (Bax) (D) were measured relative to that of GAPDH by real-time PCR analysis. Bars and error bars represent the means and standard deviations for three independent experiments. The statistical significance between the IPyr-treated group and the solvent-treated group is indicated as **P<0.01, *P<0.05, or n.s. (not significant).

Finally, we investigated the ability of the aromatic pyruvates to modulate the expression of pro-inflammatory mediators (IL-1β, IL-6, and Cox-2) and a critical pro-apoptotic regulator (Bax, a member of the Bcl-2 family of cell death regulatory proteins) in the UVB-irradiated hairless mouse skin. Among the tested compounds, only IPyr significantly attenuated UVB-induced expression of IL-1β and IL-6 mRNA (Fig. 7A, B). IPyr tended to reduce Cox-2 mRNA expression, but the effect was not significant when compared with the UVB-irradiated, solvent-treated group (Fig. 7C). These observations imply that IPyr inhibits UVB-induced production of inflammatory mediators *in vivo* as well as *in vitro*. Furthermore, IPyr significantly inhibited Bax mRNA expression in response to UVB irradiation (Fig. 7D), suggesting that IPyr also has an anti-apoptotic capacity against UVB-induced skin damage.

## Discussion

Our previous study demonstrated that pyruvate safeguards human keratinocytes from UVB-induced cell damage and inflammation.[8] The present study extended these observations by showing that all of the examined aromatic pyruvate derivatives (PPyr, HPPyr, and IPyr) reduced UVB-induced cytotoxicity in HaCaT keratinocytes, and that the aromatic pyruvates were more efficacious than the parental pyruvate compound at the same concentration (25 mM, Fig. 1).

UVB-mediated skin damage is correlated with the production of inflammatory mediators in epidermal keratinocytes, including IL-1β and IL-6.[25], [26] We found that all of the investigated aromatic pyruvates inhibited UVB-induced production of IL-1β and IL-6 in HaCaT human keratinocytes, but that IPyr was particularly effective (Figs. 2, 3A, B).

Cyclooxygenase isoenzymes (cyclooxygenase 1 (Cox-1) and Cox-2)) are rate-limiting enzymes involved in the synthesis of prostaglandins from arachidonic acid. Cox-1 is constitutively expressed in most tissues, whereas Cox-2 is induced under conditions of inflammation. The induction of Cox-2 by UVB radiation is mediated by both transcriptional and post-transcriptional mechanisms.[24] For example, p38 MAPK mediates the UVB-induced elevation of Cox-2 promoter activity in human keratinocytes.[24] p38 MAPK antagonists prevent the generation of inflammatory cytokines and the up-regulation of Cox-2 following exposure to UVB energy.[8], [27] Therefore, p38 MAPK is a critical regulator of keratinocyte responses to UVB irradiation, including inflammation and apoptosis.[23] The current study showed that IPyr inhibited the UVB-evoked activation of p38 MAPK and the up-regulation of Cox-2 expression at 5 mM (Fig. 4), suggesting that the augmented survival of IPyr-treated HaCaT cells might result from the attenuation of UVB-induced p38 MAPK signalling.

Interestingly, the effects of IPyr on UVB-irradiated keratinocytes were distinct from those of other indole derivatives, such as melatonin. Melatonin increases the survival rate of UVB-irradiated keratinocytes,[11], [12], [13], [15], [16], [17] but it must be present at the time of irradiation to exert its protective actions. By contrast, IPyr prevented UVB-induced cellular damage and up-regulation of IL-1β, IL-6, and Cox-2, even though it was added to the cell culture after UVB exposure (Figs. 2 and 3). In addition, while melatonin suppresses the inflammatory response primarily by a direct free radical-scavenging action,[13] IPyr did not inhibit ROS production in UVB-irradiated keratinocytes (Fig. S2). According to the results of our previous study, pyruvate also reduced UVB damage to keratinocytes, apart from their ROS scavenging abilites.[8] These observations suggest that the antioxidant activity of IPyr is not wholly responsible for its defensive actions in UVB-irradiated HaCaT keratinocytes. Our findings also exclude the possibility of direct UVB absorption by IPyr. Thus, the compound probably exerted its protective effects herein by regulating the expression of inflammatory and/or apoptotic mediators.

The biological significance of IPyr actions was confirmed by an *in vivo* analysis using HR-1 hairless mice. We found that topically applied IPyr ameliorated dorsal skin erythema in the UVB-irradiated mice (Fig. 5) diminished transepithelial water loss (Fig. 6) and reduced the mRNA expression of IL-1β, IL-6, Cox-2, and Bax (Fig. 7). Compared with IPyr, PPyr and HPPyr were less successful moderators of the UVB-provoked skin injury and inflammation (Figs. 5–7).

An intriguing and somewhat controversial finding of this study related to the elevation of epidermal thickness in IPyr-treated, UVB-irradiated mice (Fig. 6). The epidermis increases in thickness in response to UVB exposure in an attempt to prevent further UV damage and carcinogenesis.[28] On the other hand, epidermal hypertrophy is widely believed to be a symptom of photoaging, as it results in wrinkle formation. Other investigators showed that a p38 MAPK inhibitor blocked the UVB-induced enhancement of epidermal thickness.[29] Therefore, we anticipated that the suppression of p38 MAPK activity by IPyr would similarly decrease epidermal thickness in UVB-irradiated mice. To the contrary, IPyr-treated, UVB-irradiated mice showed an even thicker epidermis than solvent-treated, UVB-irradiated mice (Fig. 6G), suggesting that the down-regulation of p38MAPK activity might not be the only way in which IPyr can affect cell proliferation and/or survival within the skin. Future studies are required into this area of investigation to clarify the actions of IPyr.

Glucocorticoids, the final product of HPA activation during stress, have a long history of use as therapeutic agents for numerous skin disorders. Cortisol is a primary glucocorticoid and responsible for negative feedback loop in the HPA axis. [5], [30], [31], [32], [33] Proinflammatory cytokines are potent inducers of endogenous cortisol. [34] Glucocorticoid treatment of keratinocytes inhibits production of inflammatory mediators. [35] Further studies will determine the effects of indole pyruvates on the HPA axis in UVB-irradiated skin tissue.

In conclusion, the present study demonstrated, for the first time, a defensive effect of IPyr on UVB-irradiated human keratinocytes and HR-1 hairless mice skin. Moreover, IPyr showed beneficial actions *in vitro* at lower concentrations than pyruvate. Nonetheless, the UVB-protective molecular mechanism of action of IPyr remains to be elucidated.

## Supporting Information

Figure S1 **Experimental design layout.** (A) UVB-irradiation device. UVLM-28 EL UV lamp (UVP) was placed on transparent acrylic panels. (B) Stainless mouse cage for UVB-exposure. The mice were housed in specially designed cages where they were held in dividers separated by stainless steel gauze. (C) The UVB-irradiation schedule in hairless mice.
(DOCX)

Figure S2 **Effects of aromatic pyruvates on the UVB-induced production of reactive oxygen species (ROS) in cultured keratinocytes.** HaCaT cells were incubated with HBSS containing 20 µM ROS detection reagent (CellROX Green, Life Technologies) for 30 min. UVB (60 mJ/cm^2^)-irradiated cells were maintained for 30 min in the presence of solvent (culture medium) or 5 mM PPyr, HPPyr, or IPyr. After washing with PBS, cells were lysed in 1% Triton X-100. Fluoro-microplate reader was used to measure intracellular ROS levels.
(DOCX)

Table S1 **Primer sequences used for real time RT-PCR.**
(PDF)
