# Correction: Protective Effect of Indole-3-Pyruvate against Ultraviolet B-Induced Damage to Cultured HaCaT Keratinocytes and the Skin of Hairless Mice

**DOI:** 10.1371/journal.pone.0128054

**Published:** 2015-05-04

**Authors:** Reiji Aoki, Ayako Aoki-Yoshida, Chise Suzuki, Yoshiharu Takayama

There is an error in the fourth sentence of the Abstract. The correct sentence is: In addition, the dorsal skin of hairless mice (HR-1) was treated with test compounds (10 μmol) and exposed to UVB light (1 J/cm2) twice.

There is an error in the second sentence of the “UVB Irradiation of HR-1 Hairless Mice” subsection of the Materials and Methods. The correct sentence is: The dorsal skin of each mouse was treated with test compounds (10 μmol) or with the solvent control.

There is an error in the legend for Fig 5, “Aromatic pyruvates protect against UVB-induced erythema in HR-1 hairless mice.” Please view Fig 5 and its corrected legend here.

**Fig 5:**
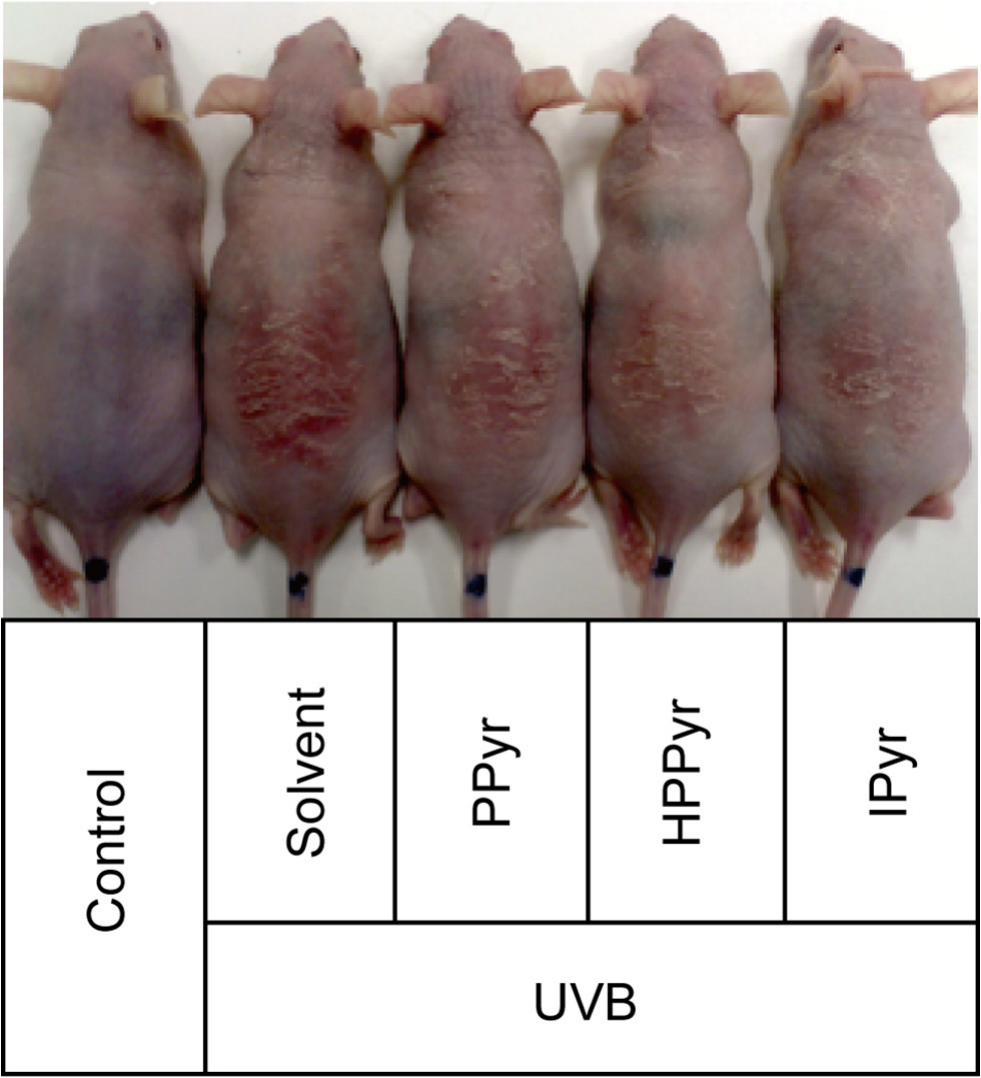
Aromatic pyruvates protect against UVB-induced erythema in HR-1 hairless mice. The dorsal skin surfaces of the hairless mice were treated with solvent (0.05 M) phosphate buffer (pH 7.0) containing 30% propylene glycol and 20% ethanol, PPyr, HPPyr, or IPyr (10 μmol), and subjected to UVB irradiation (1 J/cm2). A representative image is shown that demonstrates the appearance of the dorsal skin area in sham-irradiated (control) and UVB-irradiated mice with and without aromatic pyruvates at the end of the experiment (day 5).
