# Supplementary material for: Protective Effect of Indole-3-Pyruvate against Ultraviolet B-Induced Damage to Cultured HaCaT Keratinocytes and the Skin of Hairless Mice
Source: PLoS One. 2014 May 8;9(5):e96804. doi: 10.1371/journal.pone.0096804 (PMC4014565; doi:10.1371/journal.pone.0096804)
Supplement: Figure S1 — Experimental design layout. (A) UVB-irradiation device. UVLM-28 EL UV lamp (UVP) was placed on transparent acrylic panels. (B) Stainless mouse cage for UVB-exposure. The mice were housed in specially designed cages where they were held in dividers separated by stainless steel gauze. (C) The UVB-irradiation schedule in hairless mice. (DOCX) [file pone.0096804.s001.docx]

**
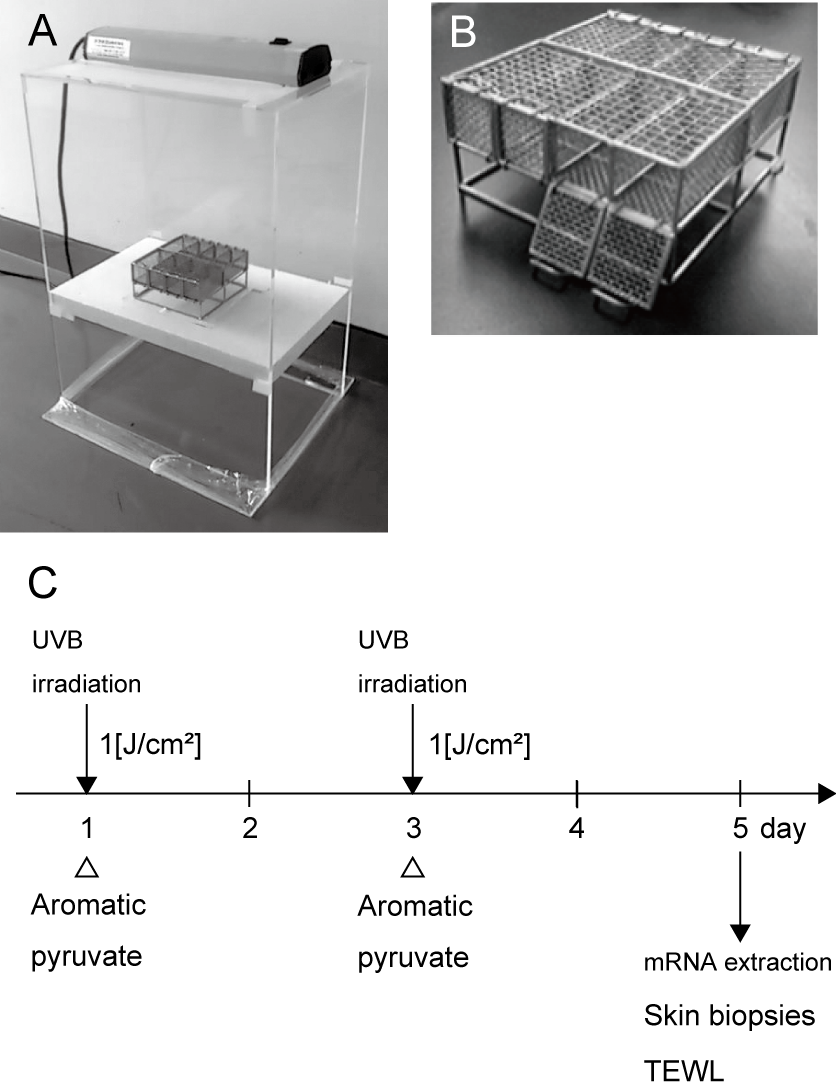
Supplementary Fig. S1.**

**Experimental design layout.** (A) UVB-irradiation device. UVM-28 EL UV lamp (UVP) was placed on transparent acrylic panels. (B) Stainless mouse cage for UVB-exposure. The mice were housed in specially designed cages where they were held in dividers separated by stainless steel gauze. (C) The UVB-irradiation schedule in hairless mice.
