# Supplementary material for: Protective Effect of Indole-3-Pyruvate against Ultraviolet B-Induced Damage to Cultured HaCaT Keratinocytes and the Skin of Hairless Mice
Source: PLoS One. 2014 May 8;9(5):e96804. doi: 10.1371/journal.pone.0096804 (PMC4014565; doi:10.1371/journal.pone.0096804)
Supplement: Figure S2 — Effects of aromatic pyruvates on the UVB-induced production of reactive oxygen species (ROS) in cultured keratinocytes. HaCaT cells were incubated with HBSS containing 20 µM ROS detection reagent (CellROX Green, Life Technologies) for 30 min. UVB (60 mJ/cm2)-irradiated cells were maintained for 30 min in the presence of solvent (culture medium) or 5 mM PPyr, HPPyr, or IPyr. After washing with PBS, cells were lysed in 1% Triton X-100. Fluoro-microplate reader was used to measure intracellular ROS levels. (DOCX) [file pone.0096804.s002.docx]

Supplemental figure 2.


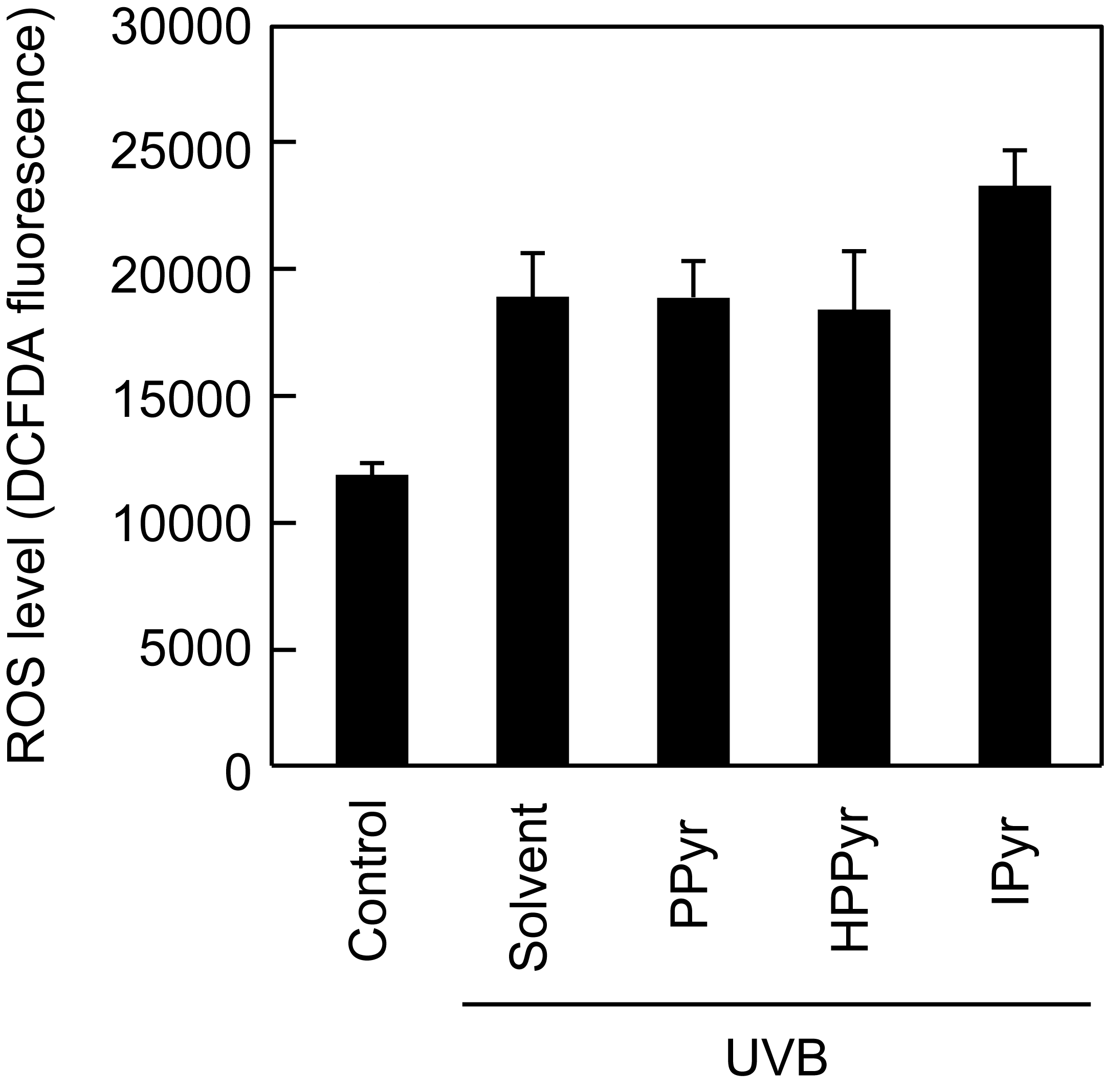


Supplementary Fig. S2.

Effects of aromatic pyruvates on the UVB-induced production of reactive oxygen species (ROS) in cultured keratinocytes. HaCaT cells were incubated with HBSS containing 20 µM ROS detection reagent (CellROX ® Green, Life Technologies) for 30 min. UVB (60 mJ/cm^2^)-irradiated cells were maintained for 30 min in the presence of solvent (culture medium) or 5 mM PPyr, HPPyr, or IPyr. After washing with PBS, cells were lysed in 1% Triton X-100. Fluoro-microplate reader was used to measure intracellular ROS levels.
