# Supplementary material for: Protective Effect of Indole-3-Pyruvate against Ultraviolet B-Induced Damage to Cultured HaCaT Keratinocytes and the Skin of Hairless Mice
Source: PLoS One. 2014 May 8;9(5):e96804. doi: 10.1371/journal.pone.0096804 (PMC4014565; doi:10.1371/journal.pone.0096804)
Supplement: Table S1 — Primer sequences used for real time RT-PCR. (PDF) [file pone.0096804.s003.pdf]

Table S1: Primer sequences used for real time RT-PCR.

Gene		Primer sequence
IL-1β primers		
IL-1β (human)	F<br>R	5’-AAGCTGATGGCCCTAAACAG -3’<br>5’-AGGTGCATCGTGCACATAAG -3’
IL-1β (mouse)	F<br>R	5’-CCTTCCAGGATGAGGACATGA -3’<br>5’-TGAGTCACAGAGGATGGGCTC -3’
IL-6 primers		
IL-6 (human)	F<br>R	5’-CCAGCTATGAACTCCTTCTC - 3’<br>5’-GCTTGTTCCTCACATCTCTC - 3’
IL-6 (mouse)	F<br>R	5’-GAGGATACCACTCCCAACAGACC -3’<br>5’-AAGTGCATCATCGTTGTTCATACA - 3’
Cox-2 primers		
Cox-2 (human)	F<br>R	5’-GAATGGGGTGATGAGCAGTT -3’<br>5’-CAGAAGGGCAGGATACAGC -3’
Cox-2 (mouse)	F<br>R	5’-CAGACAACATAAACTGCGCCTT -3’<br>5’-GATACACCTCTCCACCAATGACC -3’
GAPDH primers		
GAPDH (human)	F<br>R	5’-ACCCACTCCTCCACCTTTGA-3’<br>5’-CTGTTGCTGTAGCCAAATTCGT-3’
GAPDH (mouse)	F<br>R	5’-CCCATCACCATCTTCCAGGAGC -3’<br>5’-CCAGTGAGCTTCCCGTTCAGC -3’
Bax primer		
Bax (mouse)	F<br>R	5’-CCCGAGAGGTCTTTTTCC -3’<br>5’-GCCTTGAGCACCAGTTTG -3’
